# Skeletal Muscle Gene Expression in Nellore Cattle Fed Guanidinoacetic Acid During the Growing and Finishing Phases

**DOI:** 10.3390/ani16213353

**Published:** 2026-10-24

**Authors:** Letícia Carolina Bortolanza Soares, Leticia Kim Huang, Germán Darío Ramírez-Zamudio, Maria Eduarda Ramos, Sergio da Silva Fidelis, Andrey de Sousa Miranda, Giovana Krempel Fonseca Merighe, João Alberto Negrão, Carl Robertson Dahlen, Nara Regina Brandão Cônsolo, Saulo Luz Silva, Paulo Roberto Leme

**Affiliations:** 1 Department of Animal Nutrition and Production, School of Animal Science and Food Engineering, University of São Paulo (FZEA-USP), Pirassununga 13635-900, São Paulo, Brazil; leticiabortolanza@usp.br (L.C.B.S.); leticiahuang@usp.br (L.K.H.); mariae.ramos@usp.br (M.E.R.); sergiofidelis@usp.br (S.d.S.F.); gkrempel@usp.br (G.K.F.M.); jnegrao@usp.br (J.A.N.); sauloluz@usp.br (S.L.S.); prleme@usp.br (P.R.L.); 2 Department of Animal Science, Federal University of Lavras, Lavras 37200-900, Minas Gerais, Brazil; andrey.miranda1@estudante.ufla.br; 3 Center for Nutrition and Pregnancy and Department of Animal Sciences, North Dakota State University, Fargo, ND 58108, USA; carl.dahlen@ndsu.edu; 4 Department of Animal Nutrition and Production, College of Veterinary Medicine and Animal Science, University of São Paulo(FMVZ-USP), Pirassununga 13635-900, São Paulo, Brazil; nara.consolo@usp.br

**Keywords:** anabolic signaling, *Longissimus thoracis*, protein turnover, RT-qPCR

## Abstract

This study evaluated the effects of different levels of guanidinoacetic acid (GAA) supplementation on the transcript abundance of genes related to myogenesis, protein turnover, and energy metabolism in Nellore (*Bos indicus*) cattle during the growing and finishing phases. Fifty-one bulls were supplemented with 0, 3, 6, or 9 g GAA·kg^−1^ of supplement dry matter (DM) during the growing phase and 0, 0.3, 0.6, or 0.9 g GAA·kg^−1^ of dietary DM during the finishing phase. *Longissimus thoracis* samples were collected at the end of each phase for RT-qPCR analyses. During the growing phase, GAA supplementation increased the transcript abundance of genes associated with myogenic regulation (*PAX7*, *MYF5*, and *MYOG*) and anabolic signaling (*MTOR* and *EIF4E*), resulting in a transcriptional profile consistent with the modulation of genes involved in myogenic regulation and protein turnover, with responses differing among doses. During the finishing phase, continued GAA supplementation decreased the transcript abundance of *MYF5*, *MYOG*, *EIF4E*, *FBXO32*, and *SLC2A4*, whereas *MTOR* and *GSK3B* showed positive quadratic responses to increasing GAA doses. However, these molecular responses were not accompanied by improvements in growth performance, carcass characteristics, or most meat quality variables. Differences in gene expression observed at the end of the growing and finishing phases should be interpreted in the context of diet, management, feeding system, and duration of GAA exposure.

## Simple Summary

Guanidinoacetic acid (GAA) is a molecule involved in the production of creatine, an important energy source for muscle growth and function. This study investigated how different dietary doses of GAA affect the expression of genes related to growth and the protein and energy metabolism of skeletal muscle in Nellore (zebu) cattle during the growing and finishing phases. Fifty-one non-castrated males received 0, 3, 6, or 9 g of GAA·kg^−1^ of supplement dry matter (DM) in a protein-energy supplement during the growing phase and 0, 0.3, 0.6, or 0.9 g of GAA·kg^−1^ of dietary DM in a diet containing 75% concentrate and 25% corn silage during the finishing phase. During the growing phase, GAA supplementation altered the transcript abundance of genes associated with myogenesis, anabolic signaling, and protein turnover, with responses differing among doses. At the end of the finishing phase, continued GAA supplementation was associated with changes in the transcript abundance of genes related to myogenesis and energy metabolism. These molecular responses were not accompanied by measurable improvements in growth performance, carcass characteristics, or most meat quality traits. Overall, GAA supplementation modulated skeletal muscle gene expression, but the biological significance of these transcriptional responses requires further investigation.

## 1. Introduction

Skeletal muscle growth and development are major determinants of productive performance, carcass traits, and meat quality in beef cattle [1]. Postnatal skeletal muscle growth occurs primarily through the hypertrophy of individual muscle fibers [2], a process that depends on the activation, proliferation, and fusion of satellite cells with existing muscle fibers [3], and is regulated by a complex network of transcription factors and signaling pathways [4,5]. The expression of genes involved in these processes is influenced by factors such as age [6], breed [3], physiological status [7], nutrition [8,9], and feed additives [8]. In skeletal muscle, nutrition and feed additives directly contribute to this regulation due to the close relationship between cellular metabolism and the availability of nutrients required for tissue maintenance, growth, and remodeling [10]. Consequently, nutritional interventions targeting skeletal muscle metabolism have emerged as a promising approach to influence growth-related biological processes in beef cattle [8,11].

Guanidinoacetic acid (GAA) is an intermediate metabolite formed from the amino acids arginine and glycine [12] and is the natural precursor of creatine [13]. Guanidinoacetic acid synthesis occurs mainly in the kidney, liver, and pancreas; however, there is evidence that it may also be produced in the skeletal muscle, brain, and intestine [14,15,16,17]. Dietary supplementation with GAA has demonstrated beneficial effects on animal production by improving body weight gain [18], carcass weight gain [19], feed efficiency [20], and meat quality parameters, such as tenderness [21] and cooking loss [22]. These changes and benefits arise from the mechanism of action in which GAA serves as a creatine precursor [23], increasing energy bioavailability via the creatine-phosphocreatine system [24], and sparing amino acids such as arginine and glycine, natural precursors of GAA [12], to function in other anabolic pathways. Nevertheless, the molecular responses to GAA may vary with the animal’s physiological and developmental stage [8], as skeletal muscle undergoes significant metabolic and functional changes throughout postnatal growth [3].

During the growing phase, skeletal muscle exhibits greater plasticity, satellite-cell activity, and hypertrophic potential, whereas during the finishing phase, muscle tissue becomes physiologically more mature, with reduced growth and remodeling activity [3]. In addition, the growing and finishing phases also differ in diet, management, feeding system, and duration of GAA exposure. Recent studies have shown that GAA supplementation can modulate molecular markers involved in skeletal muscle growth, differentiation, and metabolism, including increased *IGF-1* mRNA and MHC I and IIA expression in the Longissimus muscle of beef cattle [8,25], increased *MYOD1* expression and a tendency for greater Pax-7 protein abundance, alongside reduced PAX3, in calves born to GAA-supplemented dams [26], and increased *PLIN2* expression in the pectoral muscle of Holstein cattle [11]. However, the molecular mechanisms underlying these responses remain poorly understood across postnatal developmental stages. In particular, it remains unclear whether GAA-induced changes in muscle growth and differentiation are dependent on developmental stage. Moreover, molecular evidence in *Bos indicus* cattle remains limited. Therefore, further research is needed to elucidate stage-dependent molecular responses to GAA supplementation in *Bos indicus* cattle.

Based on this knowledge gap, it was hypothesized that supplementation with increasing doses of guanidinoacetic acid would regulate the expression of genes involved in satellite-cell activity, myogenesis, protein turnover, and energy metabolism in skeletal muscle, with responses depending on the animals’ developmental stage, management system, diet, and duration of exposure to GAA. Therefore, the objective of this study was to evaluate the expression of genes related to satellite-cell activity and myogenic differentiation, anabolic signaling, proteolysis, and glucose uptake in the skeletal muscle of Nellore bulls supplemented with increasing doses of guanidinoacetic acid during the growing phase on pasture and the finishing phase in the feedlot.

## 2. Materials and Methods

This project was approved by the Ethics Committee on Animal Use of the School of Animal Science and Food Engineering of the University of São Paulo, FZEA/USP (No. 9222010623, CEUA/FZEA). All animals used in the study were selected from the herd of the Fernando Costa Campus of the University of São Paulo.

### 2.1. Animals, Period, and Experimental Design

The present study used the same Nellore bulls previously described by Soares et al. [22]. Animal performance during the growing and finishing phases, carcass traits, and meat quality data were previously published and are summarized in the Supplementary Materials (Table S7). In this manuscript, we evaluated skeletal muscle gene expression, focusing on genes involved in myogenic regulation, protein turnover, and energy metabolism, to complement the interpretation of the phenotypic responses observed by Soares et al. [22].

In summary, the experimental period was divided into two phases, growing (280 days) and finishing (94 days). During the growing period, 51 recently weaned Nellore young bulls were used (initial body weight 265 ± 19 kg; age 8 ± 2 months). The animals were allocated to paddocks with *Urochloa decumbens* pasture and allocated into blocks by body weight (BW) and randomly assigned to four treatments: control treatment, without the addition of GAA (Creamino^®^, Alzchem Trostberg GmbH, Trostberg, Germany) (CON, *n* = 13), treatment with the addition of 3 g of GAA·kg^−1^ supplement dry matter (DM) (GAA3, *n* = 13), treatment with the addition of 6 g of GAA·kg^−1^ supplement DM (GAA6, *n* = 12) and treatment with the addition of 9 g of GAA·kg^−1^ supplement DM (GAA9, *n* = 13). During the experimental period, animals received a protein-energy supplement once daily in the morning. The amount of supplement offered per paddock was calculated to provide an estimated average intake of 3 g·kg^−1^ BW per animal. The amount of supplement offered was adjusted every 28 d based on animal BW measurements. Based on the estimated supplement intake for each paddock and treatment, the estimated total daily GAA intake during the growing phase was 0, 3.4, 6.5, and 9.4 g·animal^−1^·d^−1^ for CON, GAA3, GAA6, and GAA9, respectively. The experimental area consisted of 16 paddocks, each 1.6 ha. At each rotation, 12 paddocks were used, and four remained ungrazed as reserve paddocks. Each treatment included three pre-established permanent animal groups, with four or five animals per group, and each group occupied one paddock at a time. Before each rotation, paddocks were evaluated for forage height and mass, and only those with similar grazing conditions were used. When a paddock did not meet these criteria, it was replaced by a reserve paddock. The same 12 permanent animal groups, with unchanged group composition and treatment assignment, were then randomly allocated to the eligible paddocks approximately every 56 d. Thus, only the physical paddock occupied by each group changed over time, whereas the animal group and treatment assignment remained constant. Therefore, during the growing phase, the permanent animal group, rather than the physical paddock, was considered the experimental unit. Five rotations were performed throughout the experiment (Figure S1), as described by Soares et al. [22].

In the finishing phase, all bulls (BW at feedlot entry 500.5 ± 38 kg; age 18 ± 2 months) were transferred to the experimental feedlot and allocated to three collective pens, with 17 animals per pen, according to body weight: heavy, medium, and light (Figure S2). Treatment assignment followed the experimental distribution established during the growing phase; however, within each treatment, animals were distributed across pens by BW, so each pen contained animals from all four treatments, with four or five animals per treatment in each pen. Animals remained in their assigned pens throughout the finishing phase. The finishing period included 20 d of adaptation to the feedlot facilities and experimental diets, followed by 74 d of data collection. Each pen was equipped with four electronic feeders fitted with electronic gates (Intergado^®^, Betim, MG, Brazil), with one feeder assigned to each treatment. The electronic gates allowed each animal access only to the feeder containing its assigned diet.

Because the finishing diet was offered as a total mixed ration, GAA concentrations were adjusted to target a total daily intake of the additive similar to that estimated during the growing phase. Thus, the treatments were CON, no GAA (*n* = 13); GAA3, 0.3 g GAA·kg^−1^ dietary DM (*n* = 13); GAA6, 0.6 g GAA·kg^−1^ dietary DM (*n* = 12); and GAA9, 0.9 g GAA·kg^−1^ dietary DM (*n* = 13), using a single basal diet with a corn silage-to-concentrate ratio of 25:75. Based on dry matter intake (DMI) and GAA concentration in the diet, estimated average GAA intake during the finishing phase was 0, 3.1, 5.8, and 8.9 g·animal^−1^·d^−1^ for CON, GAA3, GAA6, and GAA9, respectively.

At the end of the experimental period, all animals were slaughtered at the abattoir of the Fernando Costa Campus, University of São Paulo, in accordance with the humane slaughter guidelines established by the Regulations of the Industrial and Sanitary Inspection of Products of Animal Origin [27].

### 2.2. Commercial Source of GAA and Supplementation Procedures

The guanidinoacetic acid source used in the present study was Creamino^®^ (Alzchem Trostberg GmbH, Trostberg, Germany), batch 220324T13, a granular product containing 98.3% GAA (determined by HPLC), less than 0.1% water, and approximately 1% starch (Figure S3). The product was used within the manufacturer-reported shelf-life period. The manufacturer analytically verified the GAA concentration in the commercial product; however, no additional analyses were performed to verify its concentration in the supplements or experimental diets.

In the growing phase, GAA was incorporated into the protein-energy supplement using a horizontal mixer (Máquinas Pereira, Londrina, PR, Brazil). In the finishing phase, GAA was mixed with the concentrate at the Feed Mill of the Fernando Costa Campus, University of São Paulo, and subsequently incorporated into the total mixed ration, using a mixing time of 10 min. Ingredient composition and chemical composition of the supplements and diets are presented in Tables S1–S6, whereas intake data are presented in Table S7. No refusals associated with GAA inclusion were observed. In the growing phase, we monitored supplementation compliance collectively based on the amount of supplement offered and consumed in each paddock. In the finishing phase, we controlled access to the diet corresponding to each treatment and individual intake using electronic feeders (Intergado^®^, Betim, MG, Brazil).

### 2.3. Muscle Biopsy During the Growing Period

One week before the end of the growing phase, on day 273, biopsies were collected from the right side of the *Longissimus thoracis* muscle of all animals, between the 12th and 13th thoracic vertebrae. For gene expression analyses, a randomly selected subset of the available biopsy samples was used at the time of laboratory analysis, comprising three animals per paddock and totaling nine animals per treatment, ensuring representation of each paddock within treatments. Sampling was performed before the end of the growing phase to allow a recovery period before transfer to the feedlot, reducing possible overlap between transient responses associated with biopsy and stressors related to environmental change and regrouping into pens.

The biopsy site was previously cleaned with 70% alcohol, clipped, and infiltrated subcutaneously with lidocaine hydrochloride (Lidovet^®^, Laboratório Bravet, Rio de Janeiro, RJ, Brazil) at a dose of 0.2 mg·kg^−1^ BW, using a 2% solution (20 mg·mL^−1^), corresponding to an injection volume of 0.01 mL·kg^−1^ BW. After confirming desensitization of the anesthetized area, an incision of approximately 1 cm was made using a scalpel, and a sterile Bergström biopsy needle (Ortovet, São Paulo, SP, Brazil) was then inserted perpendicular to the muscle tissue [28]. Approximately 1 g of muscle tissue was collected from each animal, placed in cryogenic tubes, transported in liquid nitrogen, and subsequently stored in an ultra-low temperature freezer (−80 °C) for gene expression analysis. The estimated interval from tissue removal by biopsy to sample freezing in liquid nitrogen was approximately 8 min per animal.

Following the procedure, the incision site was rinsed with sterile saline solution and closed using veterinary tissue adhesive. Subsequently, a topical product with wound-healing, antibiotic, and anti-myiasis properties (Bactrovet^®^ Prata AM, König do Brasil Ltda., Jaboticabal, SP, Brazil) was applied to the incision site, and flunixin meglumine (Niglumine^®^, Ceva Saúde Animal Ltda., Paulínia, SP, Brazil) was administered intramuscularly at a dose of 1.1 mg·kg^−1^ of BW as an analgesic and anti-inflammatory agent under the guidance of the attending veterinarian. Thereafter, all animals were monitored for 72 h.

### 2.4. Muscle Collection During Slaughter

At slaughter, on day 94, following evisceration and sanitary inspection of the carcasses, samples were collected from the same nine animals per treatment previously biopsied during the growing phase. An incision of approximately 4 cm was made over the right *Longissimus thoracis* muscle between the 10th and 12th thoracic vertebrae. This location was slightly different from the biopsy site used during the growing phase to avoid the scarred area. The samples were processed on stainless steel tables, where adipose tissue and other adjacent tissues were removed, yielding approximately 1 g of muscle tissue from each animal. All sample processing was conducted on surfaces sanitized between animals using 10% chlorhexidine digluconate (Rhiorex^®^, Rioquímica S.A., São José do Rio Preto, SP, Brazil), 70% alcohol, and hydrogen peroxide (Rioquímica^®^, Rioquímica S.A., São José do Rio Preto, SP, Brazil). The samples were then snap-frozen and transported in liquid nitrogen for storage in an ultra-low temperature freezer (−80 °C) until gene expression analyses. The estimated interval from exsanguination to sample freezing in liquid nitrogen was approximately 8 min per animal.

### 2.5. Gene Expression Analysis

We evaluated eleven target genes using *ACTB* and *CASC3* as reference genes (Table 1). Primer sequences were designed based on published GenBank sequences available from the National Center for Biotechnology Information, using the *Bos indicus* genome as a reference. Amplification efficiency was evaluated for a subset of primer pairs, with efficiencies ranging from 97.8% to 109.9% and R^2^ values ranging from 0.957 to 0.997. However, complete primer-specific records of amplification efficiency, slope, and R^2^ were available only for *ACTB*, for which a standard curve yielded an efficiency of 99.789%, a slope of −3.327, and an R^2^ of 0.988. Complete records for the remaining primer pairs were unavailable.

Total RNA was extracted from approximately 50 to 100 mg of muscle tissue using TRIzol™ reagent (Invitrogen^®^, Life Technologies, Carlsbad, CA, USA), according to the manufacturer’s instructions. Samples were treated with the DNase I Amplification Grade Kit (Invitrogen^®^, Life Technologies, Carlsbad, CA, USA) to remove residual genomic DNA. RNA concentration and purity were determined using a NanoDrop OneC spectrophotometer (Thermo Fisher Scientific, Waltham, MA, USA). RNA concentrations ranged from 125.9 to 393.7 ng/µL, and A_260_/A_280_ ratios ranged from 1.75 to 1.93. RNA integrity was verified by denaturing agarose gel electrophoresis. All samples showed adequate RNA integrity, and none were excluded based on RNA quality.

For cDNA synthesis, 1 µg of total RNA was reverse-transcribed in a final volume of 20 µL using the High-Capacity cDNA Reverse Transcription Kit (Applied Biosystems™, Life Technologies, Carlsbad, CA, USA), according to the manufacturer’s instructions. Gene expression was quantified by real-time PCR using PowerUp™ SYBR™ Green Master Mix (Applied Biosystems™, Life Technologies, Carlsbad, CA, USA). Reactions were prepared in a final volume of 20 µL containing 1 µL of cDNA, 10 µL of SYBR Green Master Mix, 0.4 µM of each primer, and DEPC-treated ultrapure water to bring the reaction to final volume.

The reactions were performed using a StepOnePlus^®^ Real-Time PCR System (Applied Biosystems™, Life Technologies, Carlsbad, CA, USA), and the data were analyzed using StepOne™ Software version 2.2.2 (Applied Biosystems™, Life Technologies, Carlsbad, CA, USA). Each gene was analyzed in technical duplicate, with both replicates included on the same plate. We verified agreement between technical replicates, and no Ct values were excluded. The arithmetic mean of the duplicate Ct values was used for the analyses. For each gene and production phase, samples from all four treatments were analyzed on the same plate. A known sample was included as a positive control, and no-template controls were included in every run and showed no amplification. No reverse-transcription controls were included; however, all RNA samples were treated with DNase I before cDNA synthesis. We did not apply inter-run correction because we compared treatments separately within each production phase, with samples from all four treatments analyzed on the same plate.

The thermal protocol consisted of initial activation at 95 °C for 10 min, followed by 40 cycles of denaturation at 95 °C for 15 s and annealing and extension at 60 °C for 1 min. Melt-curve analysis from 60 °C to 95 °C was performed immediately after amplification. A single peak was observed for each primer pair, indicating no detectable nonspecific products. Table 1 presents the melting temperatures. Amplicon specificity was also verified by 1.5% agarose gel electrophoresis, which showed single bands corresponding to the expected sizes. The stability of *ACTB* and *CASC3* was assessed using geNorm during reference gene validation. The geometric mean of their expression levels was used as the normalization factor. The individual geNorm stability values (M-values) from the original analysis are no longer available and therefore are not reported. Relative transcript abundance was calculated using the 2^−ΔΔCt^ method [29]. Analyses were conducted separately for the growing and finishing phases, using the CON group within each phase as the calibrator. For each sample within each treatment, ΔΔCt was calculated relative to the mean ΔCt of the corresponding CON group (ΔΔCt = ΔCt_sample_ − mean ΔCt_CON_), and the results were expressed as fold change relative to the respective CON group [30,31].

### 2.6. Statistical Analysis

Gene expression data were analyzed using the MIXED procedure of SAS software version 9.4 (SAS Inst. Inc., Cary, NC, USA). Inferential analyses were performed using ΔCt values, whereas fold-change values calculated using the 2^−ΔΔCt^ method were used only for graphical presentation of the gene expression results. Before analysis, residual normality was assessed using the Shapiro–Wilk test, and homogeneity of variances was evaluated using Levene’s test. When the residuals did not meet the normality assumption, ΔCt values were converted to ranks using the RANK procedure in SAS, and the ranked values were analyzed using the same mixed model.

Statistical analyses were performed separately for the growing and finishing phases. During the growing phase, 12 permanent animal groups were established within three body-weight blocks, with three groups per treatment. Group composition and treatment assignment remained unchanged throughout this phase, whereas only the physical paddock occupied by each group changed during rotations. Because supplementation was provided collectively to each group, the permanent animal group was considered the experimental unit. The statistical model included treatment as a fixed effect, body-weight block as a random effect, and group nested within treatment and block as the random experimental-unit term. Initial BW in each phase was used as an adjustment covariate. For the finishing phase, the experimental unit was the individual animal because treatment intake was individually controlled using electronic feeders. The statistical model included treatment as a fixed effect and pen as a random effect. Initial body weight was included as a covariate when significant. No repeated-measures covariance structure was used because each phase was analyzed separately. A variance-components covariance structure was used for random effects. To account for the disparity in the number of experimental units among treatments, Satterthwaite’s approximation was applied to adjust the denominator degrees of freedom.

Polynomial contrasts were used to assess the linear and quadratic responses of the dependent variables to increasing treatment levels. The coefficients used for the linear and quadratic contrasts across the four GAA levels were −3, −1, 1, 3 and 1, −1, −1, 1, respectively. Least-squares means were calculated using the LSMEANS statement, and pairwise comparisons among treatments were performed using Tukey’s adjustment for multiple comparisons. Prespecified polynomial contrasts were used to assess dose–response relationships. Statistical differences were declared when *p* ≤ 0.05, and trends were discussed when 0.05 < *p* ≤ 0.10.

**Table 1:** Primer sequences (5′–3′) used in the RT-qPCR analyses.

Symbol	Gene	NCBI Code	Sequence ^1^	Amplicon	Tm (°C)	Reference
*PAX7*	*Paired box protein*	XM_015460690.2	F: GACCCTCCAGTTTCCTCATTT	106	82.30	[32]
			R: CCAGTTATGAAACCCTCCTCTG			
*MYF5*	*Myogenic factor 5*	NM_174116.1	F: AGCGTCTACTGTCCTGATGTA	99	78.87	[33]
			R: GTTGGTGATCCGATCCACTATG			
*MYOD*	*Myogenic differentiation 1*	NM_001040478.2	F: CGACGGCATGATGGACTAC	82	87.52	[34]
			R: CGCCTCGCTGTAGTAAGTGC			
*MYOG*	*Myogenin (myogenic factor 4)*	NM_001111325.1	F: CCTACAGACGCCCACAATCT	93	84.06	[35]
			R: TATGGTTTCATCTGGGAAGG			
*MTOR*	*Mammalian target of rapamycin*	XM_015475105.1	F: CATGGAAATGGCATCCAAG	90	83.64	[34]
			R: GCTCGCTTCACCTCAAACTC			
*EIF4E*	*Eukaryotic translation initiation factor 4E*	NM_174310.3	F: AAACCACCCCTACTCCGAAT	113	78.57	[36]
			R: TGCCCATCTGTTCTGTAAAGG			
*GSK3B*	*Glycogen synthase kinase 3 beta*	NM_001101310.1	F: GCCCAGAACCACCTCCTTT	95	84.23	[36]
			R: TGCTGCCATCTTTGTCTCTG			
*FBXO32*	*F-box protein 32*	NM_001046155.1	F: GAAACGCTTCCTGGACGAGA	125	82.74	[37]
			R: TCTTCTTGGCTGCAACGTCA			
*TRIM63*	*Tripartite motif containing 63*	NM_001046295	F: CCTGATCCAGGATGGAAACCC	149	85.86	[37]
			R: CAGCCTGCTGGAAGATGTCGT			
*IGF1R*	*Insulin-like growth factor 1 receptor*	HQ703508.1	F: TGCGGTTCTGTTGATAGTGG	101	79.31	[34]
			R: CAGAGGCATACAGCACTCCA			
*SLC2A4*	*Solute carrier family 2 member 4*	NM_174604.1	F: AGTATGTGGCGGATGCTATG	101	80.79	[32]
			R: TCGGGTTTCAGGCACTTT			
*ACTB*	*Actin beta*	NM_173.979.3	F: GTCCACCTTCCAGCAGATGT	90	84.07	[34]
			R: CAGTCCGCCTAGAAGCATTT			
*CASC3*	*Cancer susceptibility candidate 3*	NM_001098069.1	F: GGACCTCCACCTCAGTTCAA	85	81.84	[34]
			R: GTCTTTGCCGTTGTGATGAA			

^1^ F: forward; R: reverse.

## 3. Results

### 3.1. Phenotypic Responses to Guanidinoacetic Acid Supplementation

As previously reported by Soares et al. [22], GAA supplementation did not affect productive performance during the growing phase (*p* ≥ 0.152) or during the subsequent finishing phase, with animals remaining in the same treatment groups (*p* ≥ 0.107), carcass ultrasound measurements throughout both production phases (*p* ≥ 0.115), post-slaughter carcass characteristics (*p* ≥ 0.101), or most meat quality traits (*p* ≥ 0.107). The only exception was cooking loss, which decreased linearly with GAA supplementation (*p* = 0.015), with the lowest values observed in the GAA9 treatment compared to CON group. A brief summary can be found in the Supplementary Materials (Table S7).

### 3.2. Effects of Guanidinoacetic Acid Supplementation on Skeletal Muscle Gene Expression

**Figure 1:**
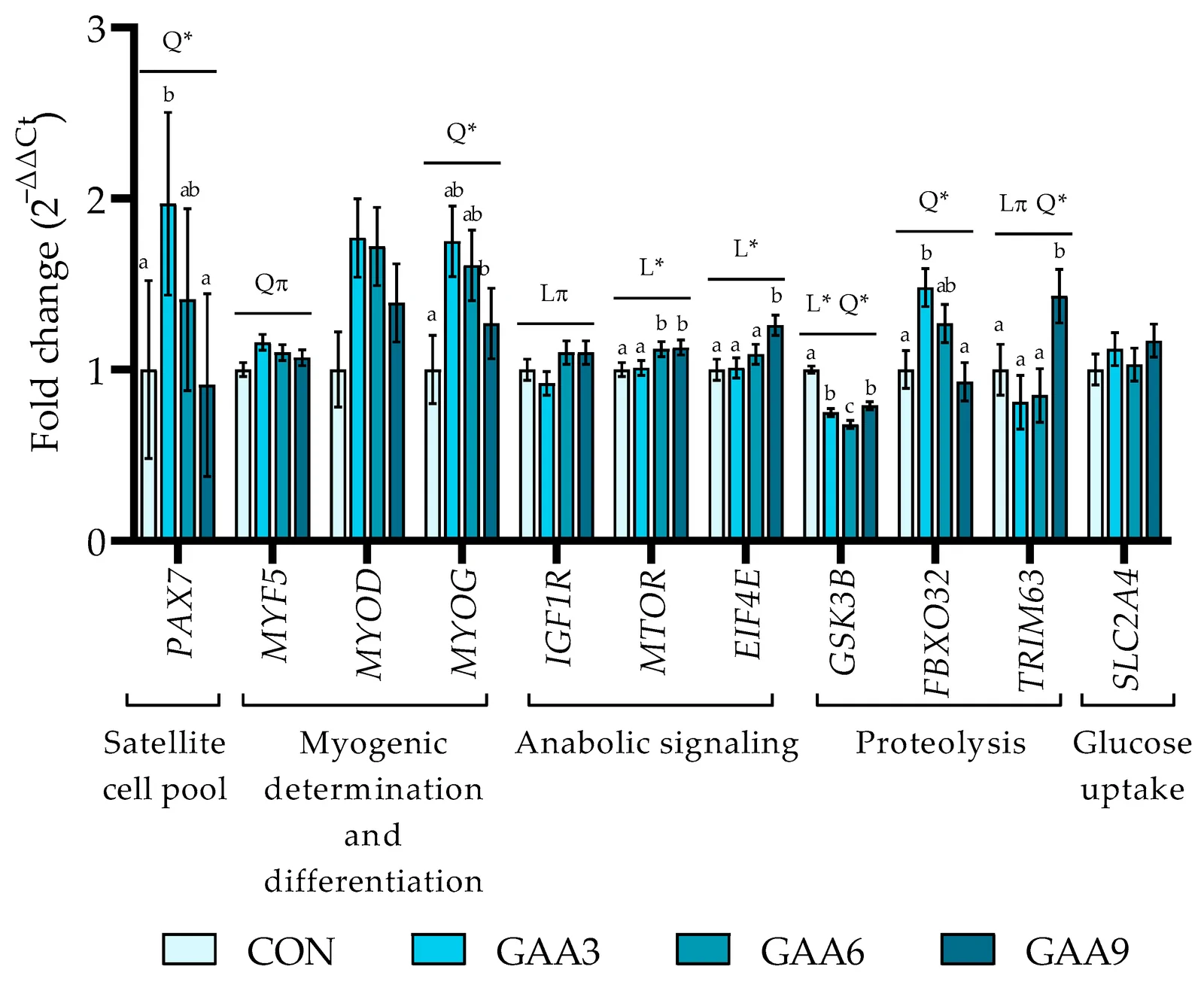
Relative expression of skeletal muscle genes in the *Longissimus thoracis* of Nellore cattle at the end of the growing phase with guanidinoacetic acid supplementation. Relative expression was calculated using the 2^−ΔΔCt^ method and shown as fold-change relative to the CON group. Bars show mean ± SEM, with *n* = 9 animals per treatment. Treatments: CON: without guanidinoacetic acid; GAA3: 3 g GAA·kg^−1^ supplement DM; GAA6: 6 g GAA·kg^−1^ supplement DM; GAA9: 9 g GAA·kg^−1^ supplement DM. Linear (L) and quadratic (Q) effects of guanidinoacetic acid supplementation were tested. Symbols indicate *p* ≤ 0.05 (*); 0.05 < *p* ≤ 0.10 (π). Different superscript letters above bars indicate differences among treatment means, whereas means sharing at least one superscript letter do not differ statistically. Complete group estimates, SEM, sample size, and exact *p*-values are provided in Supplementary Table S8. Paired box 7 (*PAX7*), myogenic factor 5 (*MYF5*), myogenic differentiation 1 (*MYOD*), myogenin (*MYOG*), insulin-like growth factor 1 receptor (*IGF1R*), mechanistic target of rapamycin (*MTOR*), eukaryotic translation initiation factor 4E (*EIF4E*), glycogen synthase kinase 3 beta (*GSK3B*), F-box protein 32 (*FBXO32*), tripartite motif containing 63 (*TRIM63*), and solute carrier family 2 member 4 (*SLC2A4*).

At the end of the growing phase (Figure 1; Table S8), a quadratic effect was observed for *PAX7* (*p* = 0.032), with greater transcript abundance in GAA3 than in CON and GAA9, whereas GAA6 showed an intermediate value and did not differ from the other treatments. The *MYF5* gene showed a trend toward a quadratic effect (*p* = 0.061), with higher transcript abundance in GAA3 compared to CON based on pairwise comparisons, whereas GAA6 and GAA9 did not differ from the other treatments. A quadratic effect was observed for *MYOG* (*p* = 0.009), with greater transcript abundance in the GAA3 compared with CON based on pairwise comparisons, whereas GAA6 and GAA9 did not differ from the other treatments. For *IGF1R*, a trend toward a linear effect was observed (*p* = 0.063), indicating a gradual increase in transcript abundance with increasing GAA dose. The *MTOR* (*p* = 0.014) and *EIF4E* (*p* = 0.004) genes exhibited linear effects, with transcript abundance increasing as GAA supplementation increased. For *MTOR*, greater values were observed in GAA6 and GAA9 than in CON and GAA3 based on pairwise comparisons, whereas the highest *EIF4E* value was observed in GAA9. For the *GSK3B* gene, both linear and quadratic effects were observed (*p* < 0.0001), with lower transcript abundance in all GAA-supplemented treatments than in CON, based on pairwise comparisons, reaching the lowest value in the GAA6 group. The *FBXO32* gene showed a quadratic effect (*p* = 0.001), with the highest transcript abundance in the GAA3 treatment, whereas GAA6 and GAA9 did not differ from the CON group. For *TRIM63*, a quadratic effect was observed (*p* = 0.049), with higher transcript abundance in the GAA9 treatment compared with the other treatments; pairwise comparisons showed that GAA6 and GAA9 did not differ from the CON group. Finally, the *MYOD* and the *SLC2A4* gene showed no treatment effect (*p* ≥ 0.102).

**Figure 2:**
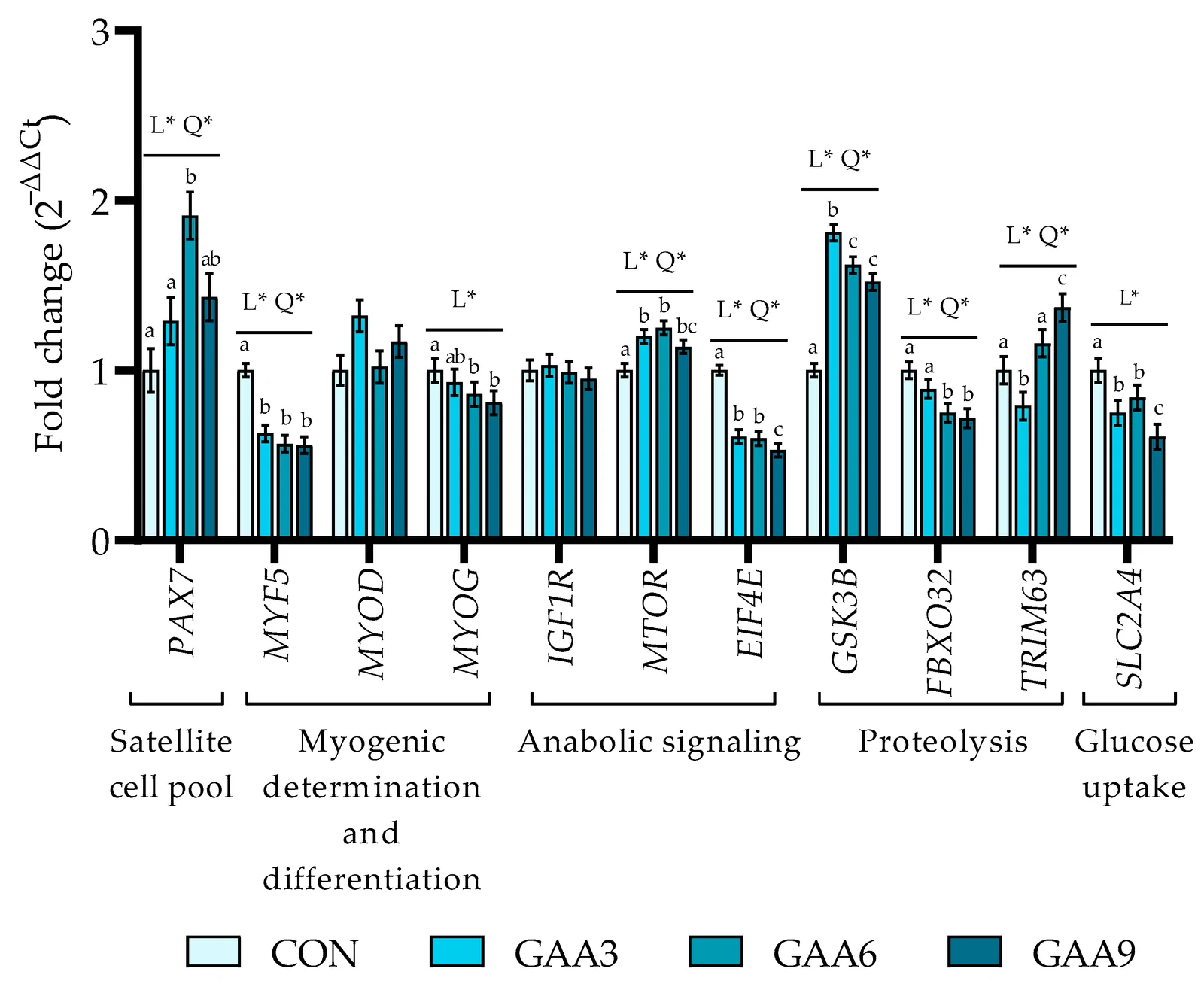
Relative expression of skeletal muscle genes in the *Longissimus thoracis* of Nellore cattle at the end of the finishing phase with guanidinoacetic acid supplementation. Relative expression was calculated using the 2^−ΔΔCt^ method and shown as fold-change relative to the CON group. Bars show mean ± SEM, with *n* = 9 animals per treatment. Treatments: CON, without guanidinoacetic acid; GAA3, 0.3 g GAA·kg^−1^ dietary DM; GAA6, 0.6 g GAA·kg^−1^ dietary DM; GAA9, 0.9 g GAA·kg^−1^ dietary DM. Linear (L) and quadratic (Q) effects of guanidinoacetic acid supplementation were tested. Symbols indicate *p* ≤ 0.05 (*); 0.05 < *p* ≤ 0.10 (π). Different superscript letters above bars indicate differences among treatment means, whereas means sharing at least one superscript letter do not differ statistically. Complete group estimates, SEM, sample size, and exact *p*-values are provided in Supplementary Table S9. Paired box 7 (*PAX7*), myogenic factor 5 (*MYF5*), myogenic differentiation 1 (*MYOD*), myogenin (*MYOG*), insulin-like growth factor 1 receptor (*IGF1R*), mechanistic target of rapamycin (*MTOR*), eukaryotic translation initiation factor 4E (*EIF4E*), glycogen synthase kinase 3 beta (*GSK3B*), F-box protein 32 (*FBXO32*), tripartite motif containing 63 (*TRIM63*), and solute carrier family 2 member 4 (*SLC2A4*).

At the end of the finishing phase, following continuous exposure to the assigned treatments across both production phases (Figure 2; Table S9), both linear (*p* = 0.003) and quadratic (*p* = 0.007) effects were observed for *PAX7*, with greater transcript abundance in GAA6 than in CON and GAA3 based on pairwise comparisons, whereas GAA9 did not differ from the other treatments. The *MYF5* gene also showed linear (*p* < 0.0001) and quadratic (*p* = 0.005) effects, with lower transcript abundance in all GAA-supplemented treatments than in CON based on pairwise comparisons. The *MYOG* gene showed a linear decrease in transcript abundance (*p* = 0.008), with lower values in GAA6 and GAA9 than in CON based on pairwise comparisons. Both linear (*p* = 0.013) and quadratic (*p* = 0.001) effects were observed for *MTOR*, with greater transcript abundance in GAA3 and GAA6 than in CON based on pairwise comparisons, whereas GAA9 did not differ from the other GAA-supplemented treatments. *EIF4E* transcript abundance also showed linear (*p* < 0.0001) and quadratic (*p* = 0.005) effects, with lower values in all GAA-supplemented treatments than in CON based on pairwise comparisons, and the lowest transcript abundance observed in GAA9 among the supplemented treatments. For the *GSK3B* gene, both linear and quadratic effects were observed (*p* < 0.0001), with greater transcript abundance in all GAA-supplemented treatments than in CON based on pairwise comparisons, reaching the highest value in GAA3. *FBXO32* showed a linear decrease in transcript abundance (*p* = 0.001), with lower values in GAA6 and GAA9 than in CON based on pairwise comparisons. The *TRIM63* gene showed both linear (*p* = 0.001) and quadratic (*p* = 0.014) effects, with lower transcript abundance in GAA3 and greater transcript abundance in GAA9 than in CON based on pairwise comparisons, whereas GAA6 did not differ from CON. The *SLC2A4* gene showed a linear effect (*p* < 0.0001), with lower transcript abundance across all GAA-supplemented treatments, and the lowest value observed in the GAA9 group. No treatment effects were detected for *MYOD* and *IGF1R* transcript abundance at the end of the finishing phase (*p* ≥ 0.364).

## 4. Discussion

Skeletal muscle is a major component of body tissue mass and plays an important role in movement, growth, protein turnover, and whole-body metabolism [38]. To sustain its energetic demands, muscle cells rely on ATP-regenerating systems, including aerobic oxidation, glycolysis, and the phosphocreatine-creatine system [39]. Among these, the phosphocreatine-creatine system provides a rapid source of ATP synthesis and recycling in muscle cells [40]. Because GAA is a direct precursor of creatine [23], its supplementation may increase phosphocreatine availability and thereby influence transcriptional responses related to muscle energy metabolism and protein turnover [41].

This improvement in cellular energy status may be particularly important for metabolically demanding processes such as muscle hypertrophy [42]. For muscle hypertrophy to occur, quiescent satellite cells must be activated and fuse with existing muscle fibers [43]. Although adult skeletal muscle exhibits cellular heterogeneity, including mature muscle fibers, fibroblasts, adipocytes, and endothelial cells, the expression of myogenic regulatory factors is frequently used as an indirect measure of satellite-cell activity [44]. At the end of the growing phase, *PAX7* showed a quadratic response to GAA supplementation, with greater transcript abundance in GAA3 than in CON and GAA9 based on pairwise comparisons, whereas GAA6 showed an intermediate value and did not differ from the other treatments. The *PAX7* gene is a key transcription factor involved in the maintenance and activation of the satellite-cell pool and in delaying differentiation during myogenesis [45,46]. Therefore, the greater abundance of the PAX7 transcript observed with GAA supplementation may indicate modulation of molecular mechanisms associated with satellite-cell activity, although satellite cells themselves were not directly evaluated in the present study. In addition to *PAX7*, genes such as *MYF5*, and *MYOG*, which are among the main myogenic regulatory factors [47], coordinate the transition between myogenic proliferation and differentiation [48]. The *MYF5* gene is closely associated with satellite-cell activation and proliferation [49], as well as with the transition between proliferation and myogenic differentiation [48]. Although *MYOD* expression was not affected during the growth phase, it plays a role in myogenic determination and differentiation, contributing to the progression of satellite cells toward a differentiated myogenic phenotype and to the subsequent formation and maturation of muscle fibers [50,51,52], whereas *MYOG* plays a fundamental role in terminal differentiation through primary myofiber formation and by facilitating the cell fusion required for the development of new muscle fibers [47]. During the postnatal period, the incorporation of nuclei derived from satellite cells into existing muscle fibers is an essential mechanism supporting muscle hypertrophy [53]. In this context, during the growing phase, animals supplemented with 3 and 6 g GAA·kg^−1^ supplement DM exhibited greater transcript abundance of *MYF5*, whereas *MYOG* showed a quadratic effect, with greater transcript abundance in GAA3 compared with the CON group. Although satellite-cell activity was not directly measured, these transcriptional changes are consistent with modulation of myogenic regulatory markers in skeletal muscle.

In addition to transcriptional markers associated with myogenic regulation, several signaling pathways are involved in the regulation of muscle protein accretion and hypertrophy [54]. In this context, genes such as *IGF1R*, *MTOR*, and *EIF4E* play key roles in regulating muscle growth through activation of protein synthesis pathways and translational efficiency [29,30,31]. During the growing phase, GAA supplementation at doses of 6 g·kg^−1^ supplement DM and above positively regulated *MTOR* expression, whereas only the 9 g·kg^−1^ supplement DM dose increased *EIF4E* expression. Additionally, *IGF1R* showed a positive linear trend in transcript abundance with increasing GAA inclusion, indicating a modest dose-related transcriptional response. The IGF-1 hormone plays a fundamental role in skeletal muscle anabolic pathways through the IGF-1–PI3K/Akt–mTOR axis [55], with its biological effects being mediated by activation of its corresponding cellular receptor (IGF1R) [56]. Once activated, the Akt pathway promotes mTOR phosphorylation, a protein recognized as one of the main regulators of muscle hypertrophy and protein synthesis, especially in response to energy and nutrient availability [57]. Downstream of mTOR, initiation factors such as EIF4E participate in translational initiation by facilitating mRNA binding to the ribosomal complex [58]. Although phosphorylation of these proteins was not assessed in the present study, changes in *MTOR* and *EIF4E* transcript abundance provide molecular evidence of modulation at the transcriptional level of genes involved in anabolic signaling.

Conversely, glycogen synthase kinase 3 beta (GSK-3β), another downstream branch of the Akt pathway, is classically inhibited during muscle hypertrophy [59]. The GSK3β protein acts as a negative regulator of protein synthesis and cellular growth by inhibiting important key translational pathways [60]. In the present study, animals supplemented with GAA showed a quadratic response, with lower *GSK3B* expression during the growing phase, with the greatest reduction observed at the 6 g·kg^−1^ supplement DM dose compared with the CON group. This pattern indicates reduced *GSK3B* transcript abundance at the transcriptional level and may be compatible with modulation of molecular components associated with negative regulation of anabolic signaling [61]. However, because GSK3β activity and phosphorylation status were not measured, reduced transcript abundance cannot be interpreted as evidence of reduced GSK3β activity or increased protein synthesis.

In contrast, *FBXO32* and *TRIM63* showed quadratic responses to GAA supplementation, with the 3 and 9 g·kg^−1^ supplement DM doses positively regulating their transcript abundance, respectively. FBXO32 targets specific proteins for degradation through the ubiquitin–proteasome pathway [62], whereas TRIM63 primarily acts in the proteolysis of structural sarcomeric proteins, such as α-actin, troponins, and myosin heavy chains [63]. Although these genes are classically associated with muscle atrophy, the concomitant changes in proteolytic markers, together with greater transcript abundance of *MTOR* and *EIF4E* in GAA-supplemented animals, may suggest a transcriptional pattern compatible with modulation of muscle protein remodeling [64]. However, protein-level, flux, or degradation measurements would be required to determine whether these transcriptional changes translate into increased protein turnover [65].

Given that GAA acts as a direct precursor of creatine and is involved in creatine metabolism [66,67,68], supplementation could potentially influence cellular energy metabolism and, consequently, processes related to tissue accretion. In the present study, however, such a phenotypic response was not observed during the growing phase [22]. Although GAA supplementation modulated the transcript abundance of several genes related to myogenic and anabolic pathways, these transcriptional responses were not accompanied by measurable improvements in growth performance during the growing phase.

At the end of the finishing phase, following continuous exposure to the assigned treatments across both production phases, *PAX7* and *MYF5* transcript abundance showed a quadratic response to GAA dose, whereas *MYOG* decreased linearly, and *MYOD* was unaffected. Greater *PAX7* expression was observed in animals receiving the GAA6 treatment, concomitantly with the downregulation of *MYF5* and *MYOG* in all groups supplemented with the additive. Satellite cells undergoing self-renewal and maintaining quiescence can be identified by evaluating molecular transcript abundance patterns, classically using the relationship between *PAX7* and the myogenic determination factors *MYF5* or *MYOD* [45]. In the present study, the increase in *PAX7* transcript abundance, concomitant with reductions in *MYF5* and *MYOG* in animals supplemented with continued GAA during the finishing phase, may indicate reduced activity of proliferative and terminal differentiation processes. This scenario contrasts with that observed during the growing phase, in which supplemented animals exhibited greater abundance of transcripts compatible with satellite-cell activation, proliferation, and differentiation.

Studies evaluating the effects of GAA on muscle growth-related gene expression in ruminants are still limited [8]. These studies demonstrated that GAA supplementation can modulate molecular markers involved in skeletal muscle growth, differentiation, and metabolism, including increased mRNA expression of *IGF-1* and *MHC I* and *IIA* in muscle of beef cattle [8,27]. Nevertheless, the biological effects of this additive are directly related to creatine metabolism and the maintenance of cellular energy metabolism [69]. From this perspective, studies conducted in young animals have demonstrated that creatine supplementation can increase the expression of myogenic regulatory factors, although this response progressively declines as animals reach greater physiological maturity [70]. Therefore, the lower transcript abundance of *MYF5* and *MYOG* observed at the end of the finishing phase should be interpreted as a transcriptional pattern consistent with reduced myogenic activity after continuous exposure to the assigned treatments across both production phases, in the context of greater physiological maturity in the animals.

In agreement with this interpretation, the isolated transcriptional stimulus on the *MTOR* gene at the low and intermediate doses (0.3 g GAA·kg^−1^ dietary DM and 0.6 g GAA·kg^−1^ dietary DM) was counterbalanced downstream by the concomitant increase in transcript abundance of the molecular repressor *GSK3B* and by the negative regulation of *EIF4E* and *FBXO32* compared with animals in the CON group. Collectively, the results for *MTOR*, *GSK3B*, and *EIF4E* may indicate reduced activation of mechanisms associated with protein synthesis and muscle growth in the finishing phase, a pattern consistent with the physiological decline in growth rate in cattle approaching physiological maturity [71]. In this context, although GAA supplementation modulated the transcript abundance compatible with myogenesis and anabolic signaling, these molecular changes were not reflected in final body weight or carcass muscling traits in the finishing phase evaluated by Soares et al. [22] in these same animals.

The *TRIM63* gene, traditionally recognized as a marker associated with the degradation of sarcomeric proteins [63], showed greater transcript abundance in animals supplemented with 0.9 g GAA·kg^−1^ dietary DM. Soares et al. [22], when evaluating meat quality in these same animals, observed a linear reduction in cooking losses as dietary GAA inclusion increased, with the lowest value recorded in the GAA9 group. The concurrent occurrence of these responses suggests that changes in structural protein remodeling may be a potential mechanism linking muscle transcriptional responses to meat water-holding capacity [72]. However, this interpretation remains hypothetical, because myofibrillar protein degradation, protein abundance, and structural organization were not evaluated. Therefore, the association between greater *TRIM63* transcript abundance and reduced cooking loss should be interpreted as an observation requiring further investigation rather than evidence of a causal relationship.

The differences observed between the growing and finishing phases in molecular markers compatible with myogenesis, protein synthesis, and protein degradation may be consistent with changes in skeletal muscle biology during animal development. However, these phase-dependent responses should be interpreted within the broader biological and experimental context of the study. In addition to physiological maturation, the transition from growing to finishing occurred together with changes in the production system, including the shift from pasture to feedlot, changes in diet and management, longer exposure to GAA, and differences in the sampling procedure. According to Picard et al. [3], young animals exhibit intense muscle growth associated with lower oxidative metabolism and greater glycolytic potential, whereas physiological maturity is accompanied by reduced intensity of tissue development and alterations in muscle fiber type composition, including a proportional decrease in glycolytic type IIX fibers and an increase in oxidative type I and IIA fibers. Thus, developmental changes provide an important biological context for the distinct transcriptional patterns observed between phases, but they should not be considered the sole explanation for these responses.

These metabolic and structural modifications in muscle tissue may directly influence the dynamics of the creatine-phosphocreatine system. According to MacLaren [24], fast glycolytic fibers contain approximately 30% more phosphocreatine and exhibit greater creatine kinase activity than slow oxidative fibers. However, phosphocreatine recycling and renewal occurs more rapidly in oxidative fibers due to greater oxygen availability and the high mitochondrial density characteristic of these fibers [24]. Therefore, the differences observed between the growing and finishing phases may be related to the predominance of a more glycolytic metabolism in young animals and the greater demand for rapid ATP regeneration during this stage of development, whereas in the finishing phase, the transcriptional responses to GAA may be more closely associated with genes involved in muscle energy metabolism. Moreover, differences in diet, management, duration of GAA exposure, and sampling procedures between phases may also have contributed to the distinct responses observed.

Additionally, a linear reduction in *SLC2A4* gene expression was observed with increasing dietary GAA inclusion. The *SLC2A4* gene encodes the glucose transporter GLUT4, the main protein responsible for glucose uptake in skeletal muscle [73]. Previous studies with pigs and broilers have demonstrated that GAA supplementation increases intramuscular concentrations of creatine, phosphocreatine, and ATP, and exerts a glycogen-sparing effect on muscle glycogen stores [66,67,68]. In this context, the downregulation of SLC2A4 observed in the present study may indicate transcriptional modulation of glucose transport-related pathways, since *SLC2A4* encodes GLUT4, an insulin-responsive glucose transporter in skeletal muscle [74]. However, glucose uptake and muscle creatine or phosphocreatine concentrations were not measured in the present study. Therefore, the functional relevance of the lower *SLC2A4* transcript abundance remains uncertain and should not be interpreted as direct evidence of reduced glucose uptake or altered phosphocreatine availability. Although GAA supplementation modulated the transcript abundance of genes related to metabolic regulation and energy metabolism, these transcriptional responses were not accompanied by measurable improvements in performance and carcass traits during the finishing phase. Nevertheless, additional studies integrating gene expression, protein abundance, muscle fiber composition, and metabolic analyses will be necessary to comprehensively elucidate the mechanisms through which GAA supplementation modulates skeletal muscle development and metabolism across different physiological stages of beef cattle production.

## 5. Conclusions

Supplementation with guanidinoacetic acid during the growing phase modulated the transcript abundance of genes associated with myogenesis and pathways related to protein synthesis and degradation, indicating dose-dependent responses. In the finishing phase, continuous exposure to guanidinoacetic acid modulated the transcript abundance of genes involved in metabolic regulation and energy metabolism, with responses varying according to GAA inclusion level. Collectively, these findings demonstrate that GAA supplementation modulated skeletal muscle gene expression, with distinct transcriptional patterns observed at the end of the growing and finishing phases. However, because the production phases differed in diet, management conditions, duration of GAA exposure, and sampling procedures, these differences cannot be attributed exclusively to physiological stage or muscle development.

## 6. Study Limitations

The present study has limitations that should be considered when interpreting the results. The responses evaluated at the end of the finishing phase reflect continuous exposure to the treatments during both the growing and finishing phases and, therefore, should not be interpreted as effects of an independent intervention restricted to the finishing phase. The phases also differed in diet, management, and sampling procedure, with muscle biopsy during the growing phase and post-mortem collection at the end of the finishing phase. Thus, the differences observed between phases may reflect a combination of production-related, physiological, and methodological factors and cannot be attributed exclusively to the developmental stage of the animals. However, within each phase, experimental and sampling procedures were standardized across treatments, supporting the comparisons performed at each sampling time. Gene expression analyses were performed using a randomly selected subset of nine animals per treatment. No formal a priori statistical power analysis was conducted to determine this sample size, which may have limited the ability to detect smaller responses. The lack of complete amplification efficiency data for all primer pairs is an additional methodological limitation. Although amplicon specificity was assessed, the available records do not allow full verification of comparable amplification efficiencies between the target and reference genes, as assumed by the 2^−ΔΔCt^ method. This limitation should be considered when interpreting the relative expression estimates. Finally, the study was restricted to evaluating transcript abundance and did not include analyses of protein abundance or phosphorylation, glucose uptake, myofibrillar protein degradation, muscle structural organization, satellite-cell activity, protein turnover, or muscle creatine and phosphocreatine concentrations. Therefore, the results should be interpreted as transcriptional-level responses, without assuming corresponding changes at the protein, metabolic, or functional level.

## Data Availability

All relevant data are presented in the article. The raw data are not publicly available because they are part of a dataset that will be used in additional integrative analyses with other datasets from the project.

## Supplementary Materials

The following supporting information can be downloaded at: https://www.mdpi.com/article/10.3390/ani16213353/s1, Figures S1–S3: experimental design and GAA source; Tables S1–S9: diet composition, phenotypic responses, and gene expression results [75,76,77].

## Abbreviations

The following abbreviations are used in this manuscript:

*EIF4E*	Eukaryotic translation initiation factor 4E
*IGF1R*	Insulin-like growth factor 1 receptor
*CASC3*	Cancer susceptibility candidate 3
*SLC2A4*	Solute carrier family 2 member 4
*GSK3B*	Glycogen synthase kinase 3 beta
*MTOR*	Mammalian target of rapamycin
*MYOG*	Myogenin (myogenic factor 4)
*TRIM63*	Tripartite motif containing 63
*MYOD*	Myogenic differentiation 1
ATP	Adenosine Triphosphate
GAA	Guanidinoacetic acid
*PAX7*	Paired box protein
*MYF5*	Myogenic factor 5
*FBXO32*	F-box protein 32
Q	Quadratic effect
L	Linear effect
DM	Dry matter
*ACTB*	Actin beta
CON	Control
