# Supplementary material for: Skeletal Muscle Gene Expression in Nellore Cattle Fed Guanidinoacetic Acid During the Growing and Finishing Phases
Source: Animals (Basel). 2026 Oct 24;16(21):3353. doi: 10.3390/ani16213353 (PMC13762148; doi:10.3390/ani16213353)
Supplement: Supplementary file 1 [file animals-16-03353-s001.zip › animals-4470601-supplementary.pdf]

SUPPLEMENTARY MATERIAL

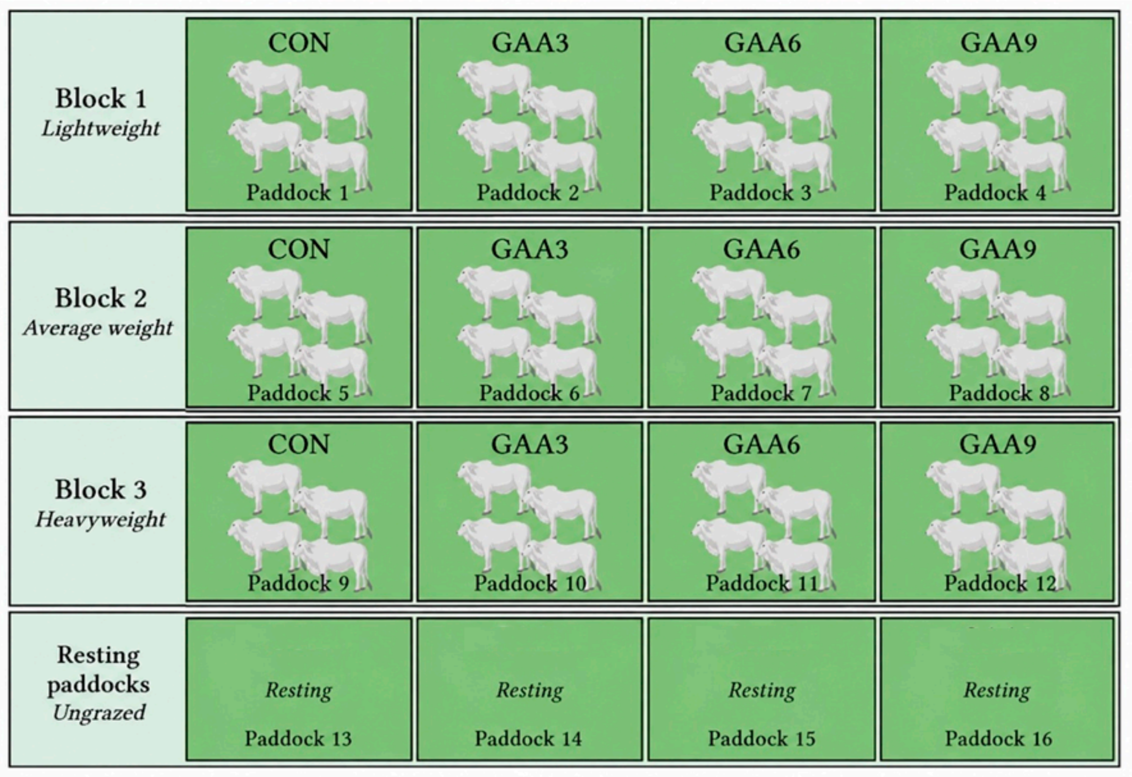

Figure S1. Experimental distribution of animals and treatments during the pasture-based growing phase. Animals were allocated to three body weight blocks and randomly assigned within each block to four treatments, resulting in three independent groups per treatment, with four or five animals per group. The experimental area consisted of 16 paddocks, of which 12 were occupied and four remained ungrazed and available for rotation. Animal groups and treatment assignments remained unchanged throughout the experiment. When forage availability became limited, the entire group was randomly moved to an available paddock, and physical paddocks were not permanently assigned to treatments. Treatments were no guanidinoacetic acid (CON), 3 g GAA·kg^{-1} supplement DM (GAA3), 6 g GAA·kg^{-1} supplement DM (GAA6), and 9 g GAA·kg^{-1} supplement DM (GAA9).

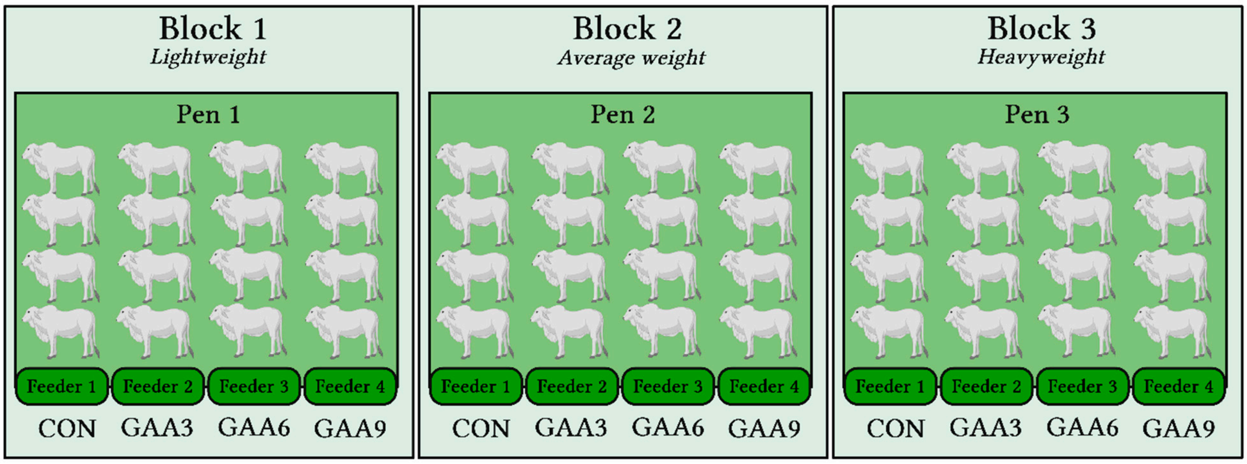

Figure S2. Illustration of the distribution of animals and treatments without the addition of guanidinoacetic acid (CON) and with the addition of guanidinoacetic acid at 0.3 g GAA·kg^{-1} dietary DM (GAA3), 0.6 g GAA·kg^{-1} dietary DM (GAA6), and 0.9 g GAA·kg^{-1} dietary DM (GAA9) during the feedlot finishing period.

alzchem group

Alzchem Trostberg GmbH - Postfach 12 62 - 83303 Trostberg - Germany

NutriQuest Nutricao Animal LTDA
Rua Comendador
Torlogo Dauntre, 74 Sala 905
CAMBUI, CAMPINAS, SAO PAULO
13025-270
BRAZIL

Inspection Certificate **PAGE 1 OF 1**
Inspection Certificate according to DIN EN 10204-3.1

Date:
14.04.2022
Purchase order No.
250
Order No. / Delivery No.
32257180 / 42228674
Goods recipient no. / Customer
0000204273

**Batch**	**220324T13**
**Quantity**	**15,000 TO**
**Production date**	2022-03-24
**Expiration date**	2025-03-24
Customer Material No.	50010601

Product: CREAMINO

**property** [method]	specification values	unit	value
**Guanidino acetic acid** HPLC	**>= 96,0**	**%**	**98,3**
**Water** Karl Fischer titration	**<= 1,0**	**%**	**< 0,1**

CREAMINO usually contains approx. 1 % starch
Alzchem Trostberg GmbH

Report machine-made; valid without signature.

The results based on our measures for quality assurance are in accordance with our specification. They do not guarantee the suitability for the product for its intended use.
For further questions regarding the mentioned figures please contact the sender.

This document is intended only for the use of the above mentioned addresses. It is confidential and may contain legally privileged information. Unauthorized reading, copying, disclosure or use is prohibited. If you received this fax through an error, please destroy your copy and inform us immediately.

Alzchem Trostberg GmbH · Head office: Trostberg · **Executive Board: Andreas Niedermaier, Klaus Engimaier, Dr. Georg Weichselbaumer**
Chairman of Supervisory Board: Markus Zöllner · **District Court Traunstein, Commercial Register No. HRB 26599 · VAT No. DE814722959**

Figure S3. Manufacturer’s inspection certificate for the commercial guanidinoacetic acid product (Creamino®, batch 220324T13) used in the experiment.

Table S1. Composition of the supplement used during the growing phase.

Ingredients (g/kg)	Suplement[1]			
	CON	GAA3	GAA6	GAA9
Ground corn	584	581	578	575
Soybean meal (45%)	350	350	350	350
Urea	25.0	25.0	25.0	25.0
Sodium chloride	20.0	20.0	20.0	20.0
Mineral Premix	21.0	21.0	21.0	21.0
Guanidinoacetic acid	-	3.0	6.0	9.0

[1] CON, without the addition of guanidinoacetic acid; GAA3, addition of 3 g/kg DM; GAA6, addition of 6 g/kg DM; GAA9 addition of 9 g/kg DM.

[2] Minerthal 160MD: Ca=230g, P= 160g, S= 60g, Co= 160mg, Cu= 270mg, F= 1600mg, I= 135mg, Mn= 2700mg, Se= 80mg, Zn= 8100mg, Monensina sódica= 4000mg.

Table S2. Composition of the final diet used during the finishing phase.

**Ingredients (g/kg)**	**Final diet[1]**			
	**CON**	**GAA3**	**GAA6**	**GAA9**
Corn silage	247.96	247.96	247.96	247.96
Ground corn	648.90	645.90	642.90	649.90
Soybean meal (45%)	69.67	69.67	69.67	69.67
Urea	11.67	11.67	11.67	11.67
Mineral Premix[2]	20.00	20.00	20.00	20.00
Potassium chloride	1.50	1.50	1.50	1.50
Magnesium Oxide[3]	0.30	0.30	0.30	0.30
Guanidinoacetic acid	-	0.30	0.60	0.90

[1] CON, without the addition of guanidinoacetic acid; GAA3, adição de 3 g/kg de MS; GAA6, adição de 6 g/kg de MS; GAA9, tratamento com adição de 9 g/kg de MS.

[2] Guabitech: Ca= 220 g; P= 20 g; Na= 60 g; S= 30 g; Mg= 20 g; K=25 g; Co= 13.30 g; Cu= 446.2 mg; I= 28.3 mg; Mn= 49 0mg; Se= 5.37 mg; Zn= 1,332 mg; Cr= 20 mg; F= 200 mg; Mananoligossacarideo= 1,511.4 mg; *Saccharomyces cerevisae*= 2,25e^{10} UFC; Monensin sodium= 1,155 mg; Beta-glucans= 4.732mg.

[3] Phix-up = 470 g/kg of magnesium Oxide.

Table S3. Nutritional profile of *Brachiaria decumbens* and the protein-energy supplement.

**Item**[1]	***Brachiaria decumbens***	**Supplement**
Nutritional profile, based on %DM		
Dry matter	37.5	89.3
Crude protein, %	5.20	31.0
Neutral detergent fiber, %	74.0	9.87
Acid detergent fiber, %	41.0	3.85
Hemicellulose, %	33.0	6.02
Ether extract, %	1.57	2.70
Ash, %	7.10	6.00
Non-fibrous carbohydrates, %	12.1	50.4
Total digestible nutrients[2], %	31.2	80.9
Digestible energy[3], Mcal/kg	1.38	3.57
Metabolizable energy[3], Mcal/kg	1.13	2.93
Net energy for maintenance[3], Mcal/kg	0.27	1.97
Net energy for gain[3], Mcal/kg	0.11	1.32

[1] Based on the nutritional profile of each ingredient, analyzed using wet chemistry procedures [1].
[2] The calculations were performed according to the equations proposed by Weiss et al. [2].
[3] Composition calculated using tabular values of NASEM [3].

Table S4. Nutritional profile of *Brachiaria decumbens* for each treatment during the growing phase.

**Item**[1]	**Controle**	**GAA3**	**GAA6**	**GAA9**
Nutritional profile, based on %DM				
Dry matter	37.3	37.7	37.7	37.0
Crude protein, %	5.30	5.40	5.40	5.00
Neutral detergent fiber, %	73.7	73.9	74.0	74.6
Acid detergent fiber, %	39.0	42.6	41.3	41.2
Hemicellulose, %	34.7	31.3	32.7	33.4
Ether extract, %	1.70	1.50	1.60	1.50
Ash, %	7.40	7.00	6.90	7.10
Non-fibrous carbohydrates, %	11.9	12.2	12.1	11.8

[1] Based on the nutritional profile of each ingredient, analyzed using wet chemistry procedures [1].

Table S5. Nutritional profile of the supplement for each treatment during the growing phase.

Item[1]	Controle	GAA3	GAA6	GAA9
Nutritional profile, based on %DM				
Dry matter	89.3	89.3	89.0	89.6
Crude protein, %	30.2	30.1	32.3	31.6
Neutral detergent fiber, %	10.2	9.50	10.0	9.80
Acid detergent fiber, %	4.00	3.90	3.70	3.80
Hemicellulose, %	6.20	5.60	6.30	6.00
Ether extract, %	2.60	2.90	2.60	2.70
Ash, %	6.40	5.90	5.80	6.10
Non-fibrous carbohydrates, %	50.6	51.6	49.3	49.8

[1] Based on the nutritional profile of each ingredient, analyzed using wet chemistry procedures [1].

Table S6. Nutritional profile of corn silage and concentrate used during the finishing of feedlot animals.

Item[1]	Corn silage	Concentrate
Nutritional profile, based on %DM		
Dry matter	30.0	87.8
Crude protein, %	9.30	17.0
Neutral detergent fiber, %	43.7	9.60
Acid detergent fiber, %	25.4	2.82
Hemicellulose, %	18.3	6.77
Ether extract, %	3.00	4.30
Ash, %	4.30	4.07
Non-fibrous carbohydrates, %	39.7	65.3
Total digestible nutrients[2], %	71.0	74.2
Digestible energy[3], Mcal/kg	3.13	3.27
Metabolizable energy[3], Mcal/kg	2.57	2.68
Net energy for maintenance[3], Mcal/kg	1.67	1.77
Net energy for gain[3], Mcal/kg	1.06	1.14

[1] Based on the nutritional profile of each ingredient, analyzed using wet chemistry procedures [1].
[2] The calculations were performed according to the equations proposed by Weiss et al. [2].
[3] Composition calculated using tabular values of NASEM [3].

Table S7. Effect of supplementation with or without GAA on the performance of bulls during the growing and finishing phases.

Item[1]	Treatments[2,3]				SEM[4]	P-value[5,6]				
	CON	GAA3	GAA6	GAA9		L	Q	Dev	Time	T×T
Growing phase										
Final BW, kg	513.4	495.2	496.2	500.4	8.31	0.316	0.192	0.654	-	-
ADG, kg/d	0.81	0.77	0.76	0.76	0.50	0.152	0.490	0.980	-	-
Finishing phase										
Final BW, kg	631.2	630.1	609.4	610.6	13.8	0.186	0.934	0.512	-	-
ADG, kg/d	1.6	1.7	1.6	1.6	0.09	0.315	0.503	0.367	-	-
DMI, kg/d	10.5	10.3	9.6	9.9	0.38	0.162	0.503	0.399	-	-
Meat quality										
CL, %	28.0	27.6	27.1	27.1	0.28	0.015	0.512	0.501	<0.001	0.887
MFI	76.3	77.1	76.4	74.5	2.24	0.523	0.556	0.982	<0.001	0.802

[1]Final BW: final body weight; ADG: average daily gain; DMI: dry matter intake; CL: cooking loss; MFI: Myofibrillar Fragmentation Index.

[2]Growing treatments= CON: No added guanidinoacetic acid (GAA); GAA3: 3 g GAA/kg supplement DM; GAA6: Addition of 6 g GAA/kg supplement DM; GAA9: Addition of 9 g GAA/kg supplement DM

[3]Finishing treatments= CON: No added guanidinoacetic acid (GAA); GAA3: Addition of 0.3 g GAA/kg DM; GAA6: Addition of 0.6 g GAA/kg DM; GAA9: Addition of 0.9 g GAA/kg DM.

[4]SEM: Standard error of the mean.

[5]L: linear effect; Q: quadratic effect; Dev: Quadratic deviation; Time: aging time; T×T: treatment × aging time interaction

[5]Complete performance and meat quality data can be accessed free of charge via the DOI: https://doi.org/10.3390/vetsci12050425.

Table S8. Relative expression of skeletal muscle genes in the *Longissimus thoracis* of Nellore cattle at the end of the growing phase with guanidinoacetic acid supplementation.

Gene (mRNA)[1]	Treatments[2,3]				p-Value[4]		
	CON	GAA3	GAA6	GAA9	Treatm.	L	Q
PAX7	1.00[a] ± 0.52	1.97[b] ± 0.53	1.41[ab] ± 0.53	0.91[a] ± 0.53	0.046	0.172	0.032
MYF5	1.00[a] ± 0.04	1.16[b] ± 0.04	1.10[ab] ± 0.04	1.07[ab] ± 0.04	0.060	0.291	0.061
MYOD	1.00 ± 0.22	1.77 ± 0.22	1.72 ± 0.23	1.39 ± 0.23	0.111	0.187	0.102
MYOG	1.00[a] ± 0.20	1.75[b] ± 0.20	1.61[ab] ± 0.21	1.27[ab] ± 0.20	0.043	0.390	0.009
IGF1R	1.00[a] ± 0.06	0.92[ab] ± 0.06	1.10[a] ± 0.07	1.10[a] ± 0.06	0.167	0.063	0.795
MTOR	1.00[a] ± 0.04	1.01[a] ± 0.04	1.12[b] ± 0.04	1.13[b] ± 0.04	0.066	0.014	0.868
EIF4E	1.00[a] ± 0.05	1.01[a] ± 0.05	1.09[a] ± 0.05	1.26[b] ± 0.05	0.002	0.004	0.117
GSK3B	1.00[a] ± 0.02	0.75[b] ± 0.02	0.68[c] ± 0.02	0.79[b] ± 0.02	<.0001	<.0001	<.0001
FBXO32	1.00[a] ± 0.11	1.48[b] ± 0.11	1.27[ab] ± 0.11	0.93[a] ± 0.10	0.001	0.348	0.001
TRIM63	1.00[a] ± 0.15	0.81[a] ± 0.17	0.85[a] ± 0.18	1.43[b] ± 0.17	0.050	0.069	0.049
SLC2A4	1.00 ± 0.09	1.12 ± 0.09	1.03 ± 0.10	1.17 ± 0.09	0.427	0.473	0.705

[1] Paired box 7 (*PAX7*), myogenic factor 5 (*MYF5*), myogenic differentiation 1 (*MYOD*), myogenin (*MYOG*), insulin-like growth factor 1 receptor (*IGF1R*), mechanistic target of rapamycin (*MTOR*), eukaryotic translation initiation factor 4E (*EIF4E*), glycogen synthase kinase 3 beta (*GSK3B*), F-box protein 32 (*FBXO32*), tripartite motif containing 63 (*TRIM63*), and solute carrier family 2 member 4 (*SLC2A4*). [2] CON: without guanidinoacetic acid; GAA3: 3 g GAA·kg^{-1} supplement DM; GAA6: 6 g GAA·kg^{-1} supplement DM; GAA9: 9 g GAA·kg^{-1} supplement DM. [3] Different superscript letters within a row indicate differences among treatment means. Means sharing at least one superscript letter do not differ statistically. [4] Overall treatment effect (Treatm.); Linear (L) and quadratic (Q) effects of guanidinoacetic acid supplementation were tested. Significant when $P \leq 0.05$ and $0.05 < P \leq 0.10$.

Table S9. Relative expression of skeletal muscle genes in the *Longissimus thoracis* of Nellore cattle at the end of the finishing phase with guanidinoacetic acid supplementation.

Gene (mRNA)[1]	Treatments[2,3]				*p*-Value[4]		
	CON	GAA3	GAA6	GAA9	Treatm.	L	Q
PAX7	1.00[a] ± 0.13	1.29[a] ± 0.13	1.91[b] ± 0.14	1.43[ab] ± 0.13	0.001	0.003	0.007
MYF5	1.00[a] ± 0.04	0.63[b] ± 0.04	0.57[b] ± 0.04	0.56[b] ± 0.04	<.0001	<.0001	0.005
MYOD	1.00 ± 0.09	1.32 ± 0.09	1.02 ± 0.09	1.17 ± 0.09	0.053	0.678	0.364
MYOG	1.00[a] ± 0.07	0.93[ab] ± 0.07	0.85[b] ± 0.07	0.80[b] ± 0.07	0.064	0.008	0.838
IGF1R	1.00 ± 0.06	1.03 ± 0.06	0.99 ± 0.06	0.95 ± 0.06	0.852	0.450	0.609
MTOR	1.00[a] ± 0.04	1.20[b] ± 0.04	1.25[b] ± 0.04	1.14[bc] ± 0.04	0.001	0.013	0.001
EIF4E	1.00[a] ± 0.03	0.61[b] ± 0.03	0.60[b] ± 0.04	0.53[c] ± 0.03	<.0001	<.0001	0.005
GSK3B	1.00[a] ± 0.04	1.81[b] ± 0.04	1.62[c] ± 0.04	1.52[c] ± 0.04	<.0001	<.0001	<.0001
FBXO32	1.00[a] ± 0.05	0.89[a] ± 0.05	0.75[b] ± 0.05	0.72[b] ± 0.05	0.003	0.001	0.479
TRIM63	1.00[a] ± 0.08	0.78[b] ± 0.08	1.16[a] ± 0.08	1.37[c] ± 0.08	0.001	0.001	0.014
SLC2A4	1.00[a] ± 0.07	0.75[b] ± 0.07	0.84[b] ± 0.07	0.61[c] ± 0.07	<.0001	<.0001	0.678

[1] Paired box 7 (*PAX7*), myogenic factor 5 (*MYF5*), myogenic differentiation 1 (*MYOD*), myogenin (*MYOG*), insulin-like growth factor 1 receptor (*IGF1R*), mechanistic target of rapamycin (*MTOR*), eukaryotic translation initiation factor 4E (*EIF4E*), glycogen synthase kinase 3 beta (*GSK3B*), F-box protein 32 (*FBXO32*), tripartite motif containing 63 (*TRIM63*), and solute carrier family 2 member 4 (*SLC2A4*). [2] CON: without guanidinoacetic acid; GAA3, 0.3 g GAA·kg^{-1} dietary DM; GAA6, 0.6 g GAA·kg^{-1} dietary DM; GAA9, 0.9 g GAA·kg^{-1} dietary DM. [3] Different superscript letters within a row indicate differences among treatment means. Means sharing at least one superscript letter do not differ statistically. [4] Overall treatment effect (Treatm.); Linear (L) and quadratic (Q) effects of guanidinoacetic acid supplementation were tested. Significant when $P \leq 0.05$ and $0.05 < P \leq 0.10$.

References

1. AOAC *Official Methods of Analyses*; 16th ed.; Washington, D.C, 1990;

2. Weiss, W.P.; Conrad, H.R.; St. Pierre, N.R. A Theoretically-Based Model for Predicting Total Digestible Nutrient Values of Forages and Concentrates. *Animal Feed Science and Technology* **1992**, *39*, 95–110, doi:10.1016/0377-8401(92)90034-4.

3. NASEM *Nutrient Requirements of Beef Cattle*; National Academies Press, 2016;
